# Slow walking speed and risk of cardiovascular events in type 2 diabetes: a systematic review

**DOI:** 10.3399/BJGPO.2025.0162

**Published:** 2026-06-03

**Authors:** Richard Baskerville, Fiona Reid, Pippa Oakeshott, Rebecca Fortescue

**Affiliations:** 1 Health and Life Sciences, Oxford Brookes University, Oxford, UK; 2 Department of Medical Statistics, King's College London, London, UK; 3 Population Health Research Institute, School of Health and Medical Sciences, City St George's, University of London, London, UK

**Keywords:** cardiovascular diseases, diabetes mellitus, systematic review

## Abstract

**Background:**

Cardiovascular disease (CVD) is the main cause of mortality in type 2 diabetes mellitus (T2DM) and detection of CVD risk is a key part of routine care. Slow walking speed is strongly correlated with CVD events in the general population.

**Aim:**

To see whether there is an association between reduced walking speed and increased CVD incidence in people with T2DM.

**Design & setting:**

Systematic review of studies of people with T2DM.

**Method:**

We searched studies in which usual walking speed was recorded, and participants were followed up for subsequent fatal and non-fatal cardiovascular events. PubMed Central, Web of Science, Cochrane Central Register of Controlled Trials, and Google Scholar were searched in December 2024. Studies were screened by two independent reviewers. Studies reporting walking speed or comparable indices and CVD outcomes in T2DM were included. Study quality was assessed using the Newcastle–Ottawa Scale. Heterogeneity of study populations prevented meta-analysis.

**Results:**

Out of 1281 studies identified, 53 full texts were retrieved and four were included, which were all of good quality. These involved 132 967 individuals with diabetes from the USA, the UK, and Japan. Mean study follow-up was 3–14 years. All four studies assessed walking speed by self-reported questionnaire and reported significant associations between reduced walking speed and increased CVD risk with risk or hazard ratios ranging from 1.18–5.88 (*P*<0.001–0.0001).

**Conclusion:**

This is the first systematic review to indicate an association between reduced walking speed and increased CVD incidence in T2DM. This association is seen across diverse populations and settings. Further research in T2DM could explore whether increasing walking speed reduces CVD risk.

## How this fits in

Reduced walking speed is associated with increased risk of cardiovascular events in the general population, but we do not know if this applies to people with type 2 diabetes mellitus. We conducted a systematic review following Preferred Reporting Items for Systematic reviews and Meta-Analyses guidelines of studies of people with T2DM in which usual walking speed was recorded and participants were followed up for subsequent fatal and non-fatal cardiovascular events. All four included studies showed significant associations (*P*<0.001–0.0001) between reduced walking speed and increased CVD risk with risk or hazard ratios ranging from 1.18–5.88 after adjustment for other CVD risk factors. All the included studies used self-perceived walk speed. Further research in people with T2DM is needed to validate different walk speed measures and assess their feasibility in clinical practice.

## Introduction

The incidence of diabetes mellitus is increasing substantially worldwide. Over the past three decades, the global burden of diabetes has rapidly increased from 30 million in 1985 to 382 million in 2014 with a projected further increase to 642 million by 2040.^1^ Cardiovascular disease (CVD) is approximately twice as common in people with type 2 diabetes mellitus (T2DM) compared with people with no diabetes, and is the main cause of death in this group.^2,3^ This figure has not changed significantly despite advances in treatment.^4^ Although routine care of diabetes focuses on prevention of CVD, current risk prediction tools lack reliability and individual discriminatory power,^5,6^ and there is a need to add factors to increase precision. The excess CVD risk of diabetes is not fully accounted for by adding diabetes to current models as a binary covariate, as is the case currently. Walking speed is increasingly recognised as an important independent predictor of CVD risk. Adding walk speed into general models might also explain some of this excess risk within people with T2DM.^7^

In general population cohorts, self-reported brisk habitual walking pace is associated with reduced risk of cardiorespiratory and cancer outcomes^8^ and all-cause mortality.^9^ Conversely, slow walking speeds (<1.0 metres per second [m/s]) are strongly associated with increased mortality.^10^ Indeed, the strength of association has been referred to as the ‘sixth vital sign’, after blood pressure, respiratory rate, pulse, temperature, and oxygen saturation.^11^ However, it is not clear if slow walking speed is also predictive of CVD events in people with T2DM as diabetes has complications that may affect walk speed.^12^ Diabetes-induced complications— such as neuropathy, myopathy, and vasculopathy — may interfere with the walk speed and CVD relationship, altering the correlation with CVD risk. It is therefore necessary to study diabetes-specific populations to establish the presence of the walk speed CVD relationship and estimate an effect size.

We conducted a systematic review of studies that investigated the association between walking speed and future risk of fatal and non-fatal CVD events in people with T2DM.

## Method

The systematic review protocol was registered before the review (PROSPERO CRD42024578164). We followed the Preferred Reporting Items for Systematic reviews and Meta-Analyses (PRISMA) guidelines with the additional Synthesis Without Meta-analysis (SWiM) reporting guidelines.^13^

### Data sources and searches

A systematic literature search was conducted in PubMed Central, Web of Science, Cochrane Central Register of Controlled Trials, and Google Scholar from inception until 12 December 2024. We used keywords: walking speed, diabetes, cardiovascular, and their associated synonyms and terms (Supplementary Box S1). Citations from identified publications were screened to check for further potential studies.

### Study inclusion criteria

The inclusion criteria were as follows:

- prospective and retrospective studies that investigated the incidence rate of fatal or non-fatal CVD events in individuals with T2DM and included walking speed as a primary or secondary predictor variable;
- subtypes of incident CVD events included: coronary heart disease, myocardial infarction (MI), angina, cerebrovascular events, heart failure, and major adverse cardiovascular events.

### Exclusion criteria

The exclusion criteria were as follows:

- studies solely on type 1 diabetes or gestational diabetes mellitus;
- studies using accelerometry to provide measures of walking activity in terms of intensity, step count, or distance, but not including walking speed;
- studies not available in English.

### Study selection

Two independent authors (PO, RB) screened titles and abstracts for eligibility, followed by selected full texts. Where different studies reported on the same cohort, the study closest to the inclusion criteria was selected. Any disagreements about eligibility were resolved through discussion between reviewers.

### Data extraction and quality assessment

Study data were extracted by one investigator (RB) and cross-checked by an independent reviewer (PO). Data included author, year of publication, country, diabetes type, sex distribution, mean age (range), study design, cohort name, mean length of follow-up in years, number of participants, total cases of fatal and non-fatal CVD, method of walking speed assessment, and CVD outcome effect measure with 95% confidence interval (CI). CVD covariates included in multiple regression models were also documented.

Study quality was assessed using the Newcastle–Ottawa Scale Table 1 ^14^ by two independent assessors (RB and PO). Differences were resolved by discussion.

**Table 1.** Newcastle–-Ottawa Scale assessment of study quality of cohort studies

	Hu *et al^16^*	Tanesescu *et al^15^*	Ueno *et al^18^*	Boonpor *et al^17^*
Representativeness of the exposed cohort: truly or somewhat representative of the average person in the community	Poor: diabetes-all types, 100% female, nurses, aged <55 years	Limited: 100% male professionals with T2DM	Good: diabetes-all types, company workers, single ethnicity	Good: T2DM, single ethnicity
Selection of non-exposed population: drawn from the same community	Same cohort	Same cohort	Same cohort	Same cohort
Measurement of exposure (walking speed): secure record or structured interview	2-yearly questionnaires	2-yearly questionnaires	Questionnaire at initial health check	Self-reported question, at structured interview
Outcomes not present at start of study	Diagnoses excluded from self-report questionnaire	Diagnoses and suggestive symptoms excluded from self-report questionnaire and 1 year initial washout period	Diagnoses excluded from health records and 1 year washout period	Diagnoses excluded from health records
Comparability of study and control population on the basis of design or analysis	Same population	Same population	Same population	Same population
Assessment of outcome: independent blind assessment or record linkage	Self-report and confirmation from medical records by independent, blinded medical assessor	Self-report and confirmation from medical records	Medical records	Medical records
Sufficient length of follow-up for outcomes to occur	Strong (mean 14 years)	Strong (mean14 years)	Weak (mean 3 years)	Strong (mean 10.7 years)
Adequacy of follow-up of cohort: small numbers lost to follow-up or unlikely to introduce bias	Data completeness not specified	Data completeness not specified	Data completeness not specified	Data completeness not specified

T2DM = type 2 diabetes mellitus

### Analysis

Meta-analysis was not possible owing to important differences between study populations, such as single gender studies and differences in-walk measures and cardiovascular outcomes. Instead, individual study outcome data were examined for effect size, methods and context, and areas of between study agreement highlighted.

## Results

### Literature search results

### Overview of included studies

In total, 1281 potential publications were identified (Figure 1). From these, 53 full texts were retrieved, and four studies met the inclusion criteria. In each of the included studies, people with diabetes were identified where CVD events were the primary outcome and walking speed was recorded at baseline. These four studies were published or pre-printed between 2001 and 2024, and included 132 967 individuals with diabetes from the US, the UK, and Japan (Table 2). The time periods covered by these studies were from 1976 to 2020.

**Figure 1.**
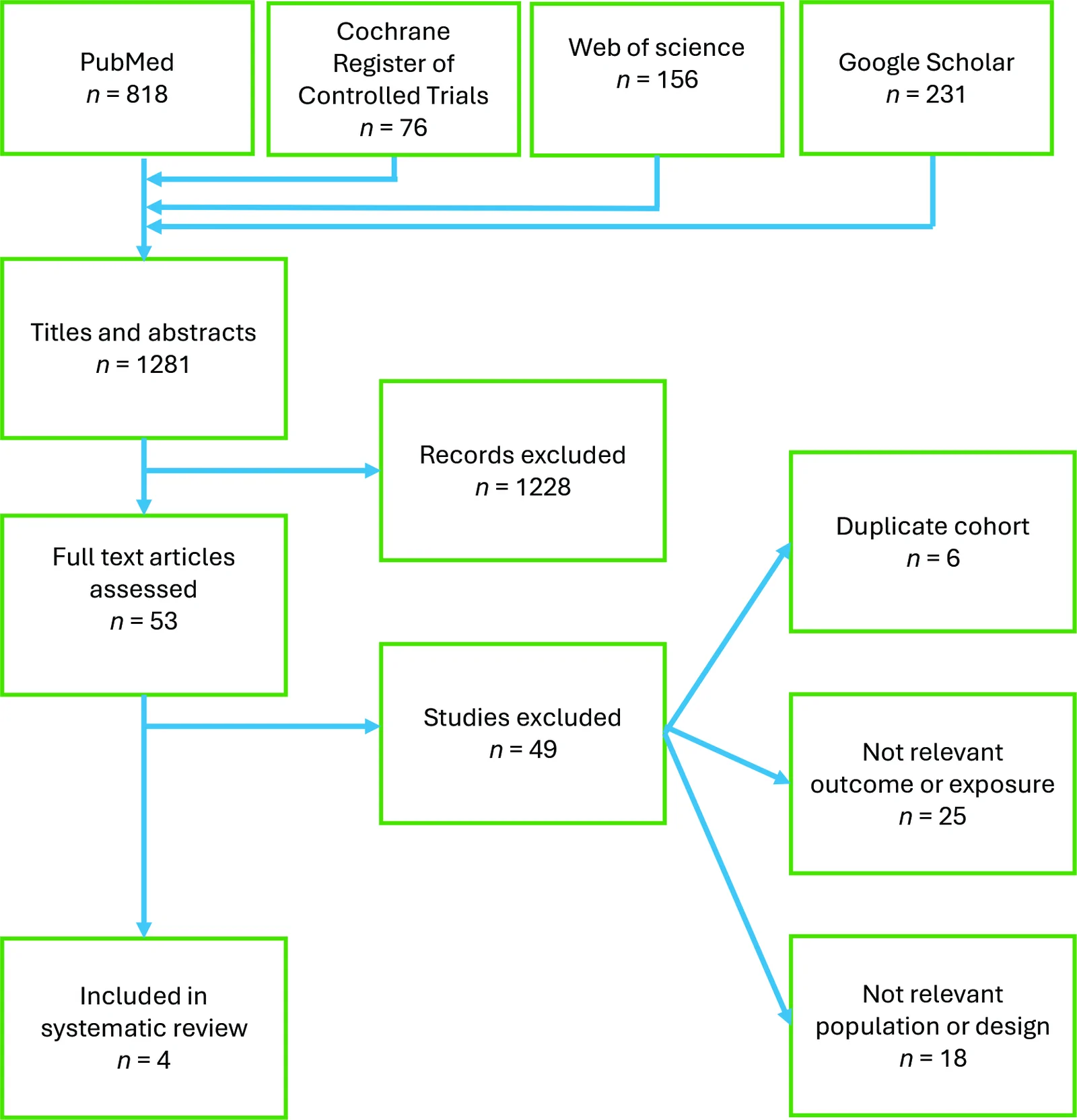
Preferred Reporting Items for Systematic reviews and Meta-Analyses (PRISMA) flowchart of included and excluded studies *25 studies excluded on the basis of not relevant predictor variables, including physical activity, step counts, and grip strength

**Table 2.** . Summary of four studies on self-reported walking speed and subsequent cardiovascular events in type 2 diabetes

Author, study site, mean follow-up period	Population cohort, study size, mean age	Composition of diabetes group	CVD outcome measure	Walking speed as primary or secondary exposure	Exposure groups: study defined group categories of walk speed	Regression model adjustment variables	Fully adjusted hazard or risk ratios
**Hu** ***et al*** **(2001),^16^** US, 14 years	The Nurses’ Health Study, 5125 females, mean age = 50 years	All diabetes types	Fatal and non-fatal CVD events	Secondary	‘Brisk’ versus ‘easy/slow’	Age, smoking, alcohol, BMI, menopausal status, vitamin supplements, hypertension, dyslipidaemia, aspirin use, diabetes duration, diabetes medication	Average: RR 0.52, brisk: RR 0.47, *P* = 0.001
**Tanesescu *et al* (2003),^15^** US, 14 years	Health Professionals Follow-up Study, 2803 males, aged 40–75 years (average age unavailable)	Type 2 diabetes	Fatal and non-fatal CVD events	Secondary	‘Normal/ brisk/striding’ versus ‘casual /slow’	Age, physical activity, alcohol, smoking, family history of CVD, vitamin E supplements, diabtes duration, diet, hypertension, cholesterol, BMI	Normal: RR 0.82, brisk: 0.58, very brisk: 0.17 (95% CI = 0.04 to 0.71; *P* = 0.001). (Inversed to give RR of slower walking: 5.88 [95% CI = 2.05 to 16.90])
**Ueno** ***et al*** **(2023)**,^18^ Japan, 3 years	Japan insurance health cohort, mixed sex, *n* = 113 065, mean age = 54 years	All diabetes types	Fatal and non-fatal CVD events	Primary	‘Normal + fast’ versus ‘slow’	Age, sex, BMI, hypertension, dyslipidaemia, physical activity, smoking	Total HR: 0.85 (95% CI = 0.811 to 0.883), HF: HR 0.839 (95% CI = 791 to 0.891), MI: HR 0.89 (95% CI = 0.773 to 1.024), angina: HR 0.892 (95% CI = 0.839 to 0.949), CVA: HR 0.793 (95% CI = 0.726 to 0.867)
**Boonpor** ***et al*** **(2024),^17^** UK, 11 years	UK Biobank, *n* = 11 974, mixed sex, mean age = 59 years	Type 2 diabetes	Fatal and non-fatal CVD events	Secondary	‘Slow’ versus ‘average/brisk’	Age, sex, deprivation, education, diet, smoking alcohol, physical activity, diabetes duration, BMI	Total cardivascular disease risk: HR 1.92 (95% CI = 1.64 to 2.24); stroke: HR 1.84 (95% CI = 1.33 to 2.55); HF: HR 2.63 (95% CI = 2.15 to 3.22); and MI: HR 1.59 (95% CI = 1.06 to 2.38)

BMI = body mass index. CI = confidence interval. CVA = cerebrovascular accident. CVD = cardiovascular disease. HF = heart failure. HR = hazard ratio. MI = myocardial infarction. RR = risk ratio

### Study design and population

The studies varied substantially in their designs and the populations studied. Tanesescu *et al*, Hu *et al*, and Boonpor *et al* were prospective cohort studies,^15–17^ while Ueno *et al* was a retrospective study based on health insurance data.^18^ Mean follow-up also varied: 3 years in Ueno *et al*, 11 years in Boonpor *et al*, and 14 years in Hu *et al* and Tanesescu *et al*.

Tanesescu *et al* ^15^ recruited 2803 male healthcare professionals aged aged 40–75 years with T2DM in the US. Hu *et al* ^16^ also focused on healthcare professionals in the US but instead recruited 5125 female nurses across 11 states with all types of diabetes. Boonpor *et al* ^17^ included 11 974 White Europeans with T2DM from the UK Biobank, a large-scale publicly accessible biomedical database. Finally, Ueno *et al* ^18^ retrospectively analysed health insurance data of 113 065 Japanese participants with all types of diabetes, following them up from their initial health check to first CVD event or study end. All four studies excluded participants with a previous history of CVD.

### Walking speed measures

All four studies relied on self-report to categorise walking speeds, using different and unvalidated scales. In Hu *et al*,^16^ participants were asked to report if their usual walking pace was *‘easy, average, brisk, or very brisk*’. Tanesescu *et al* ^15^ asked participants to categorise their walking as *‘casual, normal, brisk, or striding*’. Ueno *et al* asked participants to assess their walking speed in relation to others of the same age and sex and report if they walked faster than their peers. Finally, Boonpor *et al* ^17^ asked participants to rate their walking pace as *‘slow, steady/average or brisk*’. Tanesescu *et al* ^15^ and Hu *et al* ^16^ reported performing an objective validation test of the self-reported walking speed on a subset of participants.

### CVD outcome measures

All studies reported fatal and non-fatal CVD events and stroke. Boonpor *et al^17^* and Ueno *et al^18^* also reported heart failure, and Ueno *et al^18^* reported angina. Outcomes were collected from medical records and death certificates using international standardised definitions. Where medical records were absent or unavailable, participants were contacted individually. Completeness of follow-up data were not clearly reported in the included studies.

### Analysis

All four studies reported incident CVD events, Hu *et al^16^* and Tanesescu *et al^15^* reporting risk ratios and Ueno *et al^18^* and Boonpor *et al^17^* hazard ratios. Studies consistently adjusted their models for age, sex (in the studies recruiting both men and women), smoking, and body mass index (BMI). There were some differences in the other demographic and health variables included in the models, which are outlined in Table 2. In Ueno *et al^18^* and Hu *et al^16^* only CVD events occurring at least 1 year and 2 years, respectively, after entry into the study were counted. This reduced the risk of bias owing to reverse causality. For similar reasons, Tanesescu *et al^15^* excluded men who reported considerably reduced physical activity in the 2 years before study recruitment.

### Associations between walking speed and CVD events

All four studies reported significant associations between self-reported walking speed and CVD events after adjustment for confounding variables (*P*<0.0001–0.001). The direction of effect was consistent across studies, with slower walking speeds being associated with an increased risk of subsequent CVD events, but effect size varied substantially between studies. Tanesescu *et al*,^15^ a study including male healthcare workers in the US, found the strongest association with a more than five times risk of CVD events in the slowest walking group compared with the fastest (risk ratio 5.88, 95% confidence interval [CI] = 2.05 to 16.90) and although CIs are wide, the findings are consistent with an at least doubling of the risk. Risk or hazard ratios for the remaining studies were smaller (1.18 to 2.13) but were consistent with a significantly increased risk of CVD events in the slowest walking group (*P*<0.001). In Tanesescu *et al*, where walking was analysed in three groups, the *P* value for the trend suggests a dose-response relationship (*P*<0.001).

Two studies (Ueno *et al* and Boonpor *et al*) reported hazard ratios for specific CVD outcomes, including myocardial infarction, stroke, heart failure, and angina (Ueno *et al* only), which further confirmed the significant association between slower walking and increased cardiovascular risk (*P*<0.001; see Table 2).

Study quality, as assessed by the Newcastle–Ottawa scale, was good (Table 1).

## Discussion

### Summary

All four studies included in this systematic review found a significant association between slower walking speed and increased risk of subsequent fatal and non-fatal CVD in people with T2DM (*P*<0.001-0.0001). There was a range of effect sizes. Although there were only four studies, these were of good quality and the association was seen across diverse populations, settings, and time periods from 1976 to 2020.

### Strengths and limitations

This is the first systematic review to examine studies of walking speed as a correlate of future CVD incidence in T2DM. The prospective design of three of the four included studies, large sample sizes, long follow-up time, and confirmation through medical records contributed to the quality of evidence. All studies excluded participants with a previous history of CVD and three studies excluded participants with a recent decline in walk speed or who had experienced a CVD event early in the study, to reduce bias from possible reverse causation. We formally assessed risk of bias of included studies using a recognised tool for observational studies (Table 1).^12^ The populations included a range of ages and sexes across three continents.

However, there are limitations. Three of the included studies recruited through employment status and therefore may not be fully representative of background populations. Two studies (Hu *et al^16^* and Ueno *et al^18^*) did not differentiate between type 2 and type 1 diabetes, but owing to the approximately 9:1 ratio in prevalence, it is expected that this would not significantly change the results. Two studies (Hu *et al^16^* and Tanesescu *et al^15^*) were published more than 20 years ago, and walking speed and habits may have changed over this period. It is unclear why the mean follow-up duration in Ueno *et al* was just 3 years when the study ran from between 2005 and 2021.

There is potential for reverse causation by early cardiovascular changes influencing walk speed and later progressing to overt disease, despite studies not counting CVD in the first 1–2 years of each study period. In practice, as the walk speed and CVD relationship is likely to be circular, then the lack of causation does not affect the utility of walk speed as a predictor of CVD.

All included studies estimated walking speed by self-assessment questions, without direct measurement, and only in Ueno *et al^18^* was assessment of walking speed a primary objective of the study. To date, there is no universally standardised questionnaire and studies used constructed questions based on estimating speed compared with peers. However, this is simple to measure and is generally considered to be reliable.^19^ The assessment of walking speed by different methods prevents direct comparison or conversion to a common denominator metric. Categorisation of walking speed into a binary variable, slow versus normal or brisk, improves effect detection within a study, but only one study, Tanesescu *et al*, defined these in objective terms of miles per hour.^15^ Known differences in usual walking speeds across different age groups and countries affects study external validity. However, the peer comparison nature of self-assessment means it is unlikely that individuals were misclassified within their cohort regardless of actual speed.

### Comparison with existing literature

We found that compared with nearly 50 studies examining walking speed and CVD risk in general population cohorts,^20^ there are relatively few studies in T2DM. Indeed, only one study (Ueno *et al* ^18^), primarily examining walking speed as predictor covariate, was identified.

Our review findings in patients with T2DM are similar to those of the general population. A recent systematic review of reduced walking speed and fatal and non-fatal events showed a HR of 1.12, 95% CI = 1.09 to 1.14.^21^ This equated to a 12% increased risk of earlier mortality for each reduction of 0.1 m/s in gait speed. In more recent cohort studies using accelerometry, gait speed is calculated indirectly from physical activity intensity. This removal of self-assessment recall bias may show even larger effect sizes in CVD risk.^22^

The underlying relationship of walking speed to cardiorespiratory fitness has been well documented.^23^ Walking speed is increasingly recognised as better reflecting underlying fitness than step count or walking distance.^24–26^ Finally, in the general population, walking speed has been found to increase the predictive power of CVD multi-factor risk scores.^27^

### Implications for research and practice

Our findings indicate that walking speed in people with T2DM is related to future risk of CVD events and CVD mortality. While these studies cannot demonstrate causation, a possible explanation is that slow walking is associated with higher blood pressure, raised BMI, and impaired muscle function, which can be associated with chronic inflammation. All these markers can increase the risk of CVD. By contrast brisk walking can result in improved insulin sensitivity, glycaemic control, and lipoprotein profile,^15^ and lower BMI and blood pressure, all of which will reduce cardiovascular risk. In clinical terms, advice to patients on the benefits of brisk walking may be more important in people with T2DM who have double the CVD risk and who walk slower than people without diabetes.^12^

In general populations, the effect size of slow walking speed is comparable with those of other major CVD risk factors such as smoking and high blood pressure.^28^ Addition of walking speed to existing CVD scores in general cohorts improves CVD risk prediction.^29^ Our findings suggest that this might also apply in T2DM.

If confirmed by further research, walk speed could become an integral part of CVD risk assessment in primary care diabetes management. Assessing walking speed by a simple question might be feasible even in time-pressured clinical settings. GPs could ask: ‘Compared with other men or women your age, do you walk slower, the same, or faster?’ with slow walking speed defined as <1 m/second. Alternatively, timed-set-distance walk tests may be feasible such as 10 metre or 6 metre tests although 3 metres is also validated.^30^ Interventions to improve walking speed are known to reduce CVD mortality.^31^ Clinically recorded measures of walking speed could therefore provide both current CVD risk and inform future exercise interventions.

## Trial registration number

PROSPERO CRD42024578164
